# Reservoir computing-driven inverse dynamics for autonomous vehicle trajectory tracking control

**DOI:** 10.1038/s41598-025-32426-8

**Published:** 2025-12-18

**Authors:** Aolong Zhang, Chengyun Su, Zhifei Wang, Chao Zhou, Yuqi Yang

**Affiliations:** 1 Department of Mechanics, School of Physical Science and Engineering, Beijing Jiaotong University, Beijing, 100044 China; 2 Research Institute of Electronic Computing Technology, China Railway Research Institute Group Co., LTD, Beijing, 100044 China

**Keywords:** Trajectory tracking, Reservoir computing, Vehicle inverse dynamics, Robustness, Engineering, Mathematics and computing, Physics

## Abstract

The strong coupling between lateral and longitudinal dynamics in autonomous vehicles presents a significant challenge for trajectory tracking control, especially under high-dynamic and complex conditions. To address this, this paper proposes a real-time optimal control method driven by a Reservoir Computing (RC)-based vehicle inverse dynamics model. The approach first involves training an RC network on a comprehensive vehicle dynamics dataset, covering multiple operating conditions, to learn the inverse mapping from accelerations to control commands. Second, an online correction mechanism incorporating Proportional-Derivative (PD) feedback is designed to dynamically adjust the desired acceleration inputs based on trajectory tracking errors. Finally, these corrected accelerations are fed into the trained RC network to rapidly compute high-precision control commands, completing the closed-loop tracking. Comprehensive simulations on double-lane-change, figure-eight, and Rössler chaotic trajectories demonstrate that the proposed method achieves high-precision tracking with remarkable computational efficiency and excellent robustness against control disturbances and sensor noise. Notably, moderate sensor noise exhibits trajectory-dependent performance enhancement, with system failure boundaries under combined disturbances clearly characterized.

## Introduction

Vehicle trajectory tracking control is a core technology in autonomous driving systems, directly influencing operational safety^1^. It serves as a critical link between the planning and execution layers, translating the reference trajectory generated by the path planning module into actuator commands—such as steering, acceleration, and deceleration—to guide the vehicle smoothly along the intended path^2^. However, achieving high-precision trajectory tracking in practice presents significant challenges due to the inherent coupling of lateral and longitudinal motions in vehicle dynamics, strong nonlinearities like tire side-slip, and nonholonomic constraints^3^.

In classical trajectory tracking control architectures, lateral and longitudinal control are often managed through a decentralized framework. Independent controllers are responsible for each domain, such as the Pure Pursuit or Stanley algorithms for steering control and Proportional-Integral-Derivative (PID) or cruise control for velocity management; alternatively, separate Linear-Quadratic Regulator (LQR) or Model Predictive Control (MPC) controllers may be designed for the lateral and longitudinal tasks, respectively^4–6^. Such decoupled control strategies can achieve satisfactory performance under low-speed conditions or in scenarios where the coupling effects between lateral and longitudinal dynamics are weak. However, because these strategies neglect the mutual interactions between the lateral and longitudinal channels, the performance of decentralized control deteriorates significantly in high-dynamic scenarios that involve simultaneous aggressive steering and acceleration or deceleration^7^. Consequently, the applicability of decentralized architectures is limited, as they struggle to ensure tracking accuracy and stability under complex conditions.

To better account for the coupled characteristics of vehicle motion, centralized trajectory tracking control methods have been proposed. These approaches handle lateral and longitudinal control simultaneously within a single optimization framework. For instance, by modeling the vehicle’s lateral and longitudinal dynamics in a unified manner, methods like MPC with coupling compensation or Nonlinear MPC (NMPC) can jointly optimize the steering angle and the traction/braking forces^8,9^. Compared to decentralized strategies, centralized control can achieve superior tracking performance under conditions such as high speeds, large-curvature turns, or on low-adhesion road surfaces. However, this integrated optimization approach also introduces significant limitations. The models required to capture the vehicle’s nonlinear behavior are typically complex. Developing and calibrating these high-fidelity models is labor-intensive, increases the dimensionality of the system model and the number of optimization variables, and results in high computational costs for real-time solving, placing stringent demands on onboard computing resources^10^. Although robust control methods like Sliding Mode Control and H-infinity control exist to address model uncertainties, they often depend on expensive sensors to measure key states, such as the vehicle side-slip angle, which hinders their widespread deployment^11^.

In recent years, data-driven machine learning control methods have emerged^12^, utilizing learning algorithms to reduce the reliance on explicit models and expensive sensors. One such approach involves constructing an inverse model of the vehicle’s dynamics^13^. Typically, a neural network is employed to learn the mapping from the vehicle’s desired motion state (e.g., accelerations) to the required control inputs. A trained neural network thus represents the vehicle’s inverse dynamics, which can proactively counteract the vehicle’s internal coupling effects at the control level, achieving an approximate decoupling of the lateral and longitudinal channels. Such neural network-based inverse system control methods have shown considerable potential in simulations^14^. By introducing a learned feedforward compensation into the closed loop, for example, the accuracy of longitudinal velocity tracking can be significantly improved, and for four-wheel independent drive/steer vehicles, controllers based on this principle have been shown to reduce tracking error by correcting the reference path^15^.

However, purely data-driven control still faces several key challenges in real-world applications. First, their generalization ability is often insufficient. The performance of models like neural networks cannot be guaranteed for conditions outside their training data distribution. If a vehicle’s actual driving conditions deviate from the scope covered during training, a purely learning-based controller may suffer from decreased accuracy or even instability^16^. Second, they can lack robustness. Purely data-driven control often neglects the effects of practical factors such as sensor noise and external disturbances. In a real vehicle, sensor measurement noise and unmodeled disturbances are inevitable. A control strategy that fails to account for these can lead to trajectory deviations and even safety hazards^17,18^. Therefore, improving the generalization and disturbance-rejection robustness of learning-based trajectory tracking control has become a critical problem to be solved before this technology can be applied to real vehicles.

To address the shortcomings of existing methods, this paper proposes a vehicle trajectory tracking control framework driven by Reservoir Computing (RC). The main contributions of this work are summarized as follows:

1. **Implicit decoupling via learned inverse dynamics:** To overcome the difficulty of analytical decoupling, an RC network is trained offline to learn the vehicle’s inverse dynamics, directly mapping desired accelerations to control commands. This data-driven approach bypasses explicit coupling compensation while enabling real-time execution through a single matrix-vector multiplication, achieving 1.71$\times$–2.20$\times$ computational speedup over NMPC with 12.7%–42.0% accuracy improvement.
2. **Hybrid architecture for enhanced generalization:** To address the limited generalization of purely data-driven methods, the proposed framework combines an RC-based inverse model, which leverages rich recurrent dynamics and linear readout extrapolation, with a PD feedback loop that corrects desired acceleration inputs based on tracking errors, forming a hybrid architecture that suppresses error accumulation and maintains centimeter-level precision (10.2 mm RMSE) even on previously unseen Rössler chaotic trajectories.
3. **Systematic robustness characterization:** To quantify performance under real-world uncertainties, a comprehensive analysis under combined control disturbances and sensor noise is conducted, clearly defining the system’s failure boundaries and revealing a trajectory-dependent noise-enhancement phenomenon.

## Vehicle dynamics model and coupling effect analysis

### Dynamics modeling

The highly coupled and nonlinear characteristics of a vehicle’s lateral and longitudinal dynamics are a key bottleneck that limits the performance of traditional trajectory tracking controllers under extreme conditions. A 3-DOF (three-degree-of-freedom) model can capture the key motion characteristics of the vehicle while maintaining model simplicity, and as such, this paper establishes a 3-DOF vehicle dynamics model for its analysis^19^.

**Fig. 1:**
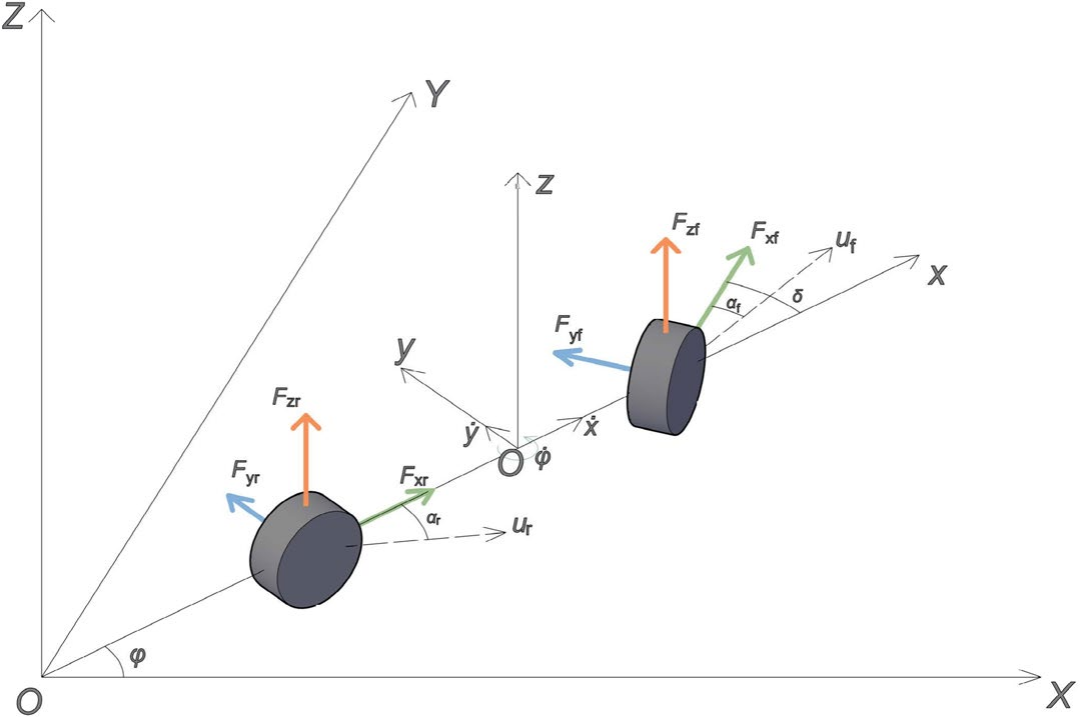
Vehicle dynamics modeling.

First, the key state variables and forces in the model are defined. In the global coordinate system (XOY), the vehicle’s motion is described by its state in the body coordinate system (xoy), which primarily includes: longitudinal velocity $\dot{x}$, lateral velocity $\dot{y}$, yaw rate $\dot{\phi }$. The terms $u_f$ and $u_r$ represent the velocity vectors at the front and rear axle centers, respectively. The vehicle’s geometric parameters are defined by the distances from the center of gravity to the front and rear axles, $l_f$ and $l_r$. The vehicle’s motion is driven and constrained by forces generated by the front and rear tires. Here, $F_{xf}$, $F_{yf}$, and $F_{zf}$ are the longitudinal, lateral, and vertical forces on the front axle tires, respectively, while $F_{xr}$, $F_{yr}$, and $F_{zr}$ are the corresponding forces on the rear axle tires. The core output of the controller is the front wheel steering angle, $\delta$, which, together with the vehicle’s yaw angle $\phi$, determines the vehicle’s trajectory. The front and rear tire side-slip angles, $\alpha _f$ and $\alpha _r$, serve as intermediate variables representing the interaction between the tires and the ground, and they directly influence the generation of lateral forces. Figure 1 illustrates the relationship between these variables in both the global and body coordinate systems.

Based on the forces and geometric relationships shown in Fig. 1, and according to Newton’s second law, the 3-DOF dynamic equations of the vehicle can be established as follows:

1 $$\begin{aligned} & m\ddot{x} = m\dot{y}\dot{\phi } + F_{xf}\cos \delta - F_{yf}\sin \delta + F_{xr} - k_h\dot{x}^2 \end{aligned}$$

2 $$\begin{aligned} & m\ddot{y} = -m\dot{x}\dot{\phi } + F_{xf}\sin \delta + F_{yf}\cos \delta + F_{yr} \end{aligned}$$

3 $$\begin{aligned} & I_z\ddot{\phi } = l_f(F_{xf}\sin \delta + F_{yf}\cos \delta ) - l_r F_{yr} \end{aligned}$$

where $m$ is the vehicle mass, $k_h$ is the air drag coefficient, and $I_z$ is the moment of inertia about the yaw axis. In vehicle control, we are more concerned with the physical quantities that can be directly measured by an onboard Inertial Measurement Unit (IMU), namely the longitudinal acceleration $a_x = \ddot{x} - \dot{y}\dot{\phi }$ and the lateral acceleration $a_y = \ddot{y} + \dot{x}\dot{\phi }$^20^.

Determining the vertical load on each axle is crucial. Based on the wheelbase $L = l_f + l_r$, the static vertical loads on the front and rear axles are $F_{zf,static} = \frac{mg l_r}{L}$ and $F_{zr,static} = \frac{mg l_f}{L}$. The vehicle’s longitudinal acceleration causes a longitudinal load transfer of $\Delta F_z = \frac{ma_x h_{cg}}{L}$, where $h_{cg}$ is the height of the vehicle’s center of gravity (CG).

Thus, the dynamic vertical loads on the front and rear axles are $F_{zf,dyn} = F_{zf,static} - \Delta F_z$ and $F_{zr,dyn} = F_{zr,static} + \Delta F_z$.

The controller generates a total longitudinal command force, $F_t$, which represents the combined traction or braking effort. This force needs to be distributed between the front and rear axles. The proportion of the longitudinal force borne by the rear axle is determined by a distribution coefficient $k_r$, which is dynamically calculated based on real-time vertical loads and whether the vehicle is accelerating or braking. First, a base distribution ratio, $k_{r,base}$, which reflects the proportion of the total dynamic vertical load on the rear axle, is calculated:

4 $$\begin{aligned} k_{r,base} = \frac{F_{zr,dyn}}{F_{zf,dyn} + F_{zr,dyn}} \end{aligned}$$

Then, an empirical upper limit is applied: $k_{r,\max } = 0.7$ for $F_t> 0$ (acceleration) and $k_{r,\max } = 0.6$ for $F_t \le 0$ (braking). The final distribution coefficient is

5 $$\begin{aligned} k_r = \min (k_{r,base}, k_{r,\max }) \end{aligned}$$

yielding the nominal longitudinal forces:

6 $$\begin{aligned} F_{xf,nom} = (1-k_r) F_t, \quad F_{xr,nom} = k_r F_t \end{aligned}$$

However, tire adhesion imposes a physical constraint. The maximum available friction force on each axle is

7 $$\begin{aligned} F_{xf,\max } = \mu F_{zf,dyn}, \quad F_{xr,\max } = \mu F_{zr,dyn} \end{aligned}$$

where $\mu$ is the road friction coefficient. To prevent wheel slip, the final applied longitudinal forces are saturated:

8 $$\begin{aligned} F_{xf} = \text {sgn}(F_{xf,nom}) \cdot \min (|F_{xf,nom}|, F_{xf,\max }), \quad F_{xr} = \text {sgn}(F_{xr,nom}) \cdot \min (|F_{xr,nom}|, F_{xr,\max }) \end{aligned}$$

This saturation logic ensures the applied force respects both the control command direction and the tire’s adhesion limit. To accurately capture the nonlinear behavior of tires under limit conditions, this model uses the Pacejka Magic Formula to calculate lateral tire forces^21^. First, the nominal lateral force parameters for the front and rear axles are defined, including the stiffness factors $B_f, B_r$, shape factors $C_f, C_r$, peak factors $D_{0f}, D_{0r}$ (at static load), and curvature factors $E_f, E_r$. With respect to the nominal peak factor under static load, $D_0$, the dynamic peak factors $D_f$ and $D_r$ can be calculated as follows:

9 $$\begin{aligned} D_f = D_{0f} \cdot \frac{F_{zf,dyn}}{F_{zf,static}}, \quad D_r = D_{0r} \cdot \frac{F_{zr,dyn}}{F_{zr,static}} \end{aligned}$$

Based on these parameters, the nominal lateral forces for the front and rear axles, $F_{yf,nom}$ and $F_{yr,nom}$, are calculated using the Pacejka Magic Formula:

10 $$\begin{aligned} F_{y,nom} = D \cdot \sin (C \cdot \arctan (B\alpha - E(B\alpha - \arctan (B\alpha )))) \end{aligned}$$

where $F_{y,nom}$ is the nominal lateral force, $\alpha$ is the tire side-slip angle, and $B, C, D, E$ are the formula parameters. This equation is applied to both the front and rear axles by substituting the respective sets of parameters (e.g., $B_f, C_f, D_f, E_f, \alpha _f$ for the front axle to compute $F_{yf,nom}$). When a tire is subjected to both longitudinal and lateral forces simultaneously, these forces compete for the total available adhesion from the ground. A friction ellipse model is used to correct the lateral force. The final actual lateral forces, $F_{yf}$ and $F_{yr}$, acting on the vehicle are calculated as follows:

11 $$\begin{aligned} & F_{yf} = F_{yf,nom} \cdot \sqrt{\max \left( 0, 1 - \left( \frac{F_{xf}}{F_{xf,\max }}\right) ^2\right) } \end{aligned}$$

12 $$\begin{aligned} & F_{yr} = F_{yr,nom} \cdot \sqrt{\max \left( 0, 1 - \left( \frac{F_{xr}}{F_{xr,\max }}\right) ^2\right) } \end{aligned}$$

The simulation parameters used in this study are detailed in Appendix A. All simulations were conducted in the MATLAB 2024b environment. For the validation phase described in the following subsection, the vehicle dynamics model was implemented through co-simulation using Simulink and CarSim. This approach leverages CarSim’s high-fidelity vehicle dynamics library to provide a more realistic representation of vehicle behavior during controller validation, while Simulink provides the control algorithm implementation and system integration environment.

### Coupling effect analysis

Based on the established dynamic model, the key coupling effects are analyzed below. To facilitate the analysis, we linearize the nonlinear Pacejka tire model around a zero side-slip angle ($\alpha =0$). At this operating point, the relationship between lateral force and side-slip angle can be approximated as linear. The proportional coefficient is defined as the effective cornering stiffness $C_{f,eff}$, which is determined by the Pacejka model parameters for the front axle: $C_{f,eff} = B_f \cdot C_f \cdot D_{f}$. The steering input, $\delta$, primarily affects the longitudinal acceleration, $a_x$, by changing the direction of the resultant force on the front wheels. When a steering angle is applied, the front-wheel longitudinal force, $F_{xf}$, which is primarily for traction or braking, generates a lateral component. Simultaneously, the lateral force, $F_{yf}$, generated to maintain the turn, produces a counteracting component along the vehicle’s longitudinal axis. Based on the longitudinal dynamics equation (Equation (1)), and assuming that a small steering input does not instantaneously change the distribution of the total longitudinal force, the sensitivity of the longitudinal acceleration to the steering angle can be derived (see Appendix B for detailed derivation):

13 $$\begin{aligned} \frac{\partial a_x}{\partial \delta } = \frac{-(F_{xf}\delta + F_{yf} + C_{f,eff}\delta )}{m} \end{aligned}$$

During normal driving, the terms in the numerator typically result in a negative value for this partial derivative. This reveals that increasing the front steering angle, $\delta$, inevitably leads to a reduction in longitudinal acceleration capability, $a_x$, due to the geometric decomposition of forces and the generation of lateral force.

In steady-state turning, the vehicle’s lateral acceleration, $a_y$, is sustained by the combined lateral forces of the front and rear wheels. When the total longitudinal command force, $F_t$, changes, it affects $a_y$ through two main pathways: 1) Load Transfer: Longitudinal acceleration or deceleration alters the vertical loads on the front and rear axles, which in turn changes the effective cornering stiffness of the tires and affects lateral force generation. 2) Tire Adhesion Competition: An increase or decrease in longitudinal force directly consumes or frees up the tire’s available adhesion. According to the friction ellipse model (Equations (11)-(12)), this either limits or enhances the tire’s ability to generate lateral force. By combining these effects and performing a linearized analysis of the steady-state force balance equations, the sensitivity of the lateral acceleration to the longitudinal force can be derived (see Appendix B for detailed derivation):

14 $$\begin{aligned} \frac{\partial a_y}{\partial F_t} = \frac{\delta (1 - k_r)}{\frac{ml_r}{L} + \frac{C_{f,eff}l_f}{v_x^2}} \end{aligned}$$

In this equation, the denominator is always positive, so the sign is determined by the numerator, $\delta$. Therefore, increasing the driving force ($F_t> 0$) will enhance the vehicle’s steering response, while applying braking force ($F_t < 0$) will weaken it.

**Fig. 2:**
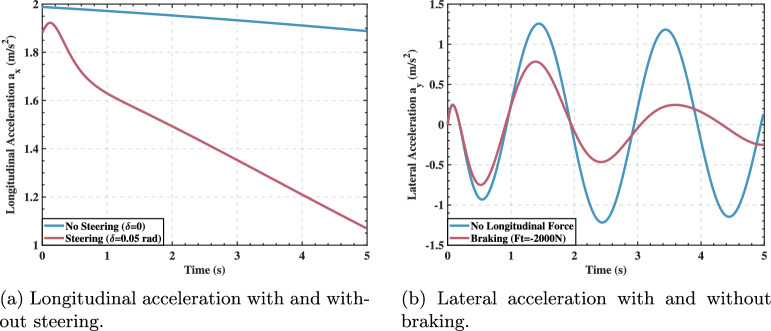
Experimental results of the coupling effect of vehicle dynamic control inputs.

To visually demonstrate these coupling effects, the simulation results in Fig. 2 clearly show that steering input significantly weakens the vehicle’s longitudinal acceleration capability, while longitudinal braking substantially reduces the vehicle’s lateral response for the same steering input.

The analysis above reveals the nonlinear coupling effects between the vehicle’s lateral and longitudinal dynamics. This is the fundamental reason why traditional control methods struggle to guarantee high precision under complex conditions. Given that decoupling schemes based on precise analytical models are difficult to implement in engineering practice, a new control paradigm is urgently needed.

## Controller design and validation based on RC inverse dynamics

This study proposes a paradigm shift in control, moving away from reliance on explicit forward modeling and analytical decoupling, and instead constructing an inverse mapping controller by learning from offline-generated data. The core idea is to directly establish an end-to-end mapping from the desired accelerations, $a_x$ and $a_y$, associated with a desired vehicle motion, to the longitudinal force $F_t$ and front steering angle $\delta$ required to produce that motion. RC provides an ideal tool to realize this concept. Theoretically, under the Echo State Property and the Separation Condition, the reservoir state space can serve as a high-dimensional embedding of the original nonlinear system state, thereby enabling reconstruction of the system dynamics^22^. In line with this, RC has been applied to robotic-manipulator trajectory tracking to demonstrate accurate and real-time performance^23^, while RC has been leveraged to tackle feedback control of nonlinear dynamical systems via online inverse-model learning^24^. As a special type of recurrent neural network, the RC’s internal reservoir, composed of high-dimensional, nonlinear neurons, is naturally adept at capturing and simulating complex temporal dynamics. We leverage this high-dimensional dynamic property, allowing the network to perform a nonlinear mapping of the coupled relationships within its feature space during learning, thereby achieving a clearer decoupled response at the output. The controller designed in this paper is based on this principle. It relies on offline-collected data of $(F_t, \delta , a_x, a_y)$ for training. During actual control, there is no need to solve complex dynamic equations; the control commands can be rapidly output with just a single matrix-vector multiplication, thus balancing the demands for high precision and high real-time performance^25,26^.

### Inverse dynamics model construction and control strategy

This paper designs a systematic data generation strategy to construct the training set by randomly exploring control inputs within physical limits. The goal is to fully excite the vehicle model and thus collect rich and diverse state response data that comprehensively reflects its internal dynamic laws. To cover different operating conditions, six initial speed groups, $v_0$ in {40, 45, 50, 55, 60, 70} km/h, are designed, focusing on mid-to-high speed conditions where vehicle dynamics coupling is most significant. The data generation process switches between these core speed groups every 1000 seconds. Within each group, the vehicle’s state is re-initialized every 80 seconds—the longitudinal velocity is reset to a value between 95% and 105% of the base speed $v_0$, while the lateral velocity $v_y$ and yaw rate $\psi$ are reset to 0—to enhance data diversity and suppress cumulative errors. For each sampling step $dt=0.001$ s, resulting in a total of $1.0 \times 10^6$ data points for training, raw control input sequences are first randomly generated within physical limits:

15 $$\begin{aligned} F_t \sim U(-3000, 3000) \, \text {N} + 250 \, \text {N}, \quad \delta \sim U(-\delta _{max}, \delta _{max}) \, \text {rad} \end{aligned}$$

where $\delta _{max} = 6.0 \times \frac{\delta _{base}}{1 + k_v v_{nom}}$ rad defines the uniform sampling range across all speed groups, with $\delta _{base} = 0.52$ rad, $k_v = 0.10$ s/m, and $v_{nom} = 15$ m/s representing the mid-range training speed. During simulation, the generated steering commands are dynamically clamped according to the instantaneous longitudinal velocity $v_x$ to ensure physical realizability:

16 $$\begin{aligned} \delta _{limit}(v_x) = \min \left( 0.992, \frac{\delta _{base}}{1 + k_v v_x}\right) \end{aligned}$$

This velocity-dependent constraint reflects the physical requirement that high-speed driving demands smaller steering angles to maintain vehicle stability. To prevent unrealistic high-frequency jitter in the randomly generated control sequences, a moving average filter with a time-based adaptive window (0.15 s) is used to smooth the inputs, yielding $F_t$ and $\delta$. To enhance model robustness, 2% control disturbances and 2% measurement noise are added to the training data. Since excessively large lateral velocity and yaw rate are physically impossible in real driving, the states $\dot{y}$ and $\dot{\phi }$ are constrained to [−5, 5] m/s and [−2, 2] rad/s, respectively. These constraints ensure that the generated data conforms to the dynamic characteristics of a real vehicle and prevent the model from learning unrealistic extreme cases.

**Fig. 3:**
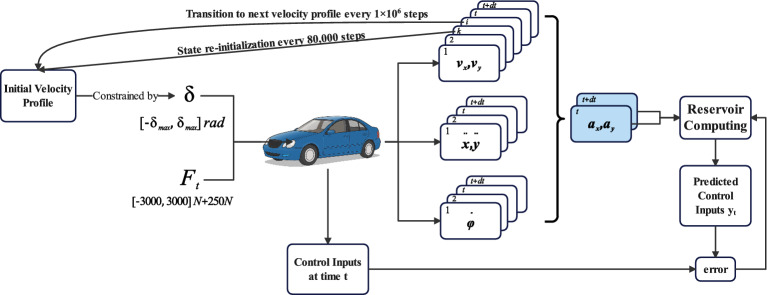
Training phase.

The training process, detailed in Fig. 3, aims to learn the vehicle’s inverse dynamics relationship. At each time step t, the randomly generated ’Control Inputs at time t’ serve a dual purpose: they drive the vehicle model to produce state and acceleration data (depicted as sequential data blocks), and they simultaneously act as the ground-truth target for the learning task. The core objective of the Reservoir Computing network is to reconstruct these control inputs from the vehicle’s acceleration response. Consequently, the training input vector $z_t$ is composed of the vehicle’s accelerations at time t and t+dt (as highlighted in blue in Fig. 3):

17 $$\begin{aligned} z_t = [a_x(t), a_y(t), a_x(t+dt), a_y(t+dt)]^\top \end{aligned}$$

The network then generates the ’Predicted Control Inputs’, $y_t$. This prediction is compared with the actual control signal applied at time t, which serves as the training target $y_{\text {target}, t} = [F_t(t), \delta (t)]^\top$, and the resulting ’error’ is used to train the network’s output weights. This closed-loop process, combined with the periodic state re-initialization described previously, enables the RC to accurately learn the complex mapping from desired accelerations to the required control commands.

Let the reservoir size be $n=200$. The internal connectivity structure determines the reservoir’s dynamics. The input weight matrix $W_{in} \in \mathbb {R}^{n \times d_{in}}$ is generated from a uniform distribution scaled by the input weight magnitude $\gamma$. The reservoir weight matrix $W \in \mathbb {R}^{n \times n}$ is constructed as a sparse symmetric matrix with density $k$ to ensure rich dynamics and the Echo State Property (ESP). Specifically, a random sparse matrix $W_{raw}$ is first generated, then rescaled so that the final matrix $W$ has spectral radius exactly $\rho$: $W = W_{raw} \cdot \frac{\rho }{|\lambda _{max}(W_{raw})|}$. The hidden state at step t is $r_t \in \mathbb {R}^n$, the leak rate is $\alpha$, and the constant bias is $k_b$. The state update equation is:

18 $$\begin{aligned} r_{t+1} = (1-\alpha )r_t + \alpha \tanh (Wr_t + W_{in}z_t + k_b\textbf{1}) \end{aligned}$$

The updated state matrix $R = [r_1^*, \dots , r_{N_{train}}^*] \in \mathbb {R}^{n \times N_{train}}$ and the target output matrix $Y_{target} = [y_{\text {target}, 1}, \dots , y_{\text {target}, N_{train}}] \in \mathbb {R}^{2 \times N_{train}}$ are used to compute the output weights $W_{out}$. The fundamental purpose of this solution process is to minimize the training error, which is the difference between the control outputs predicted by the reservoir network based on the hidden state $r_t$ (i.e., $y_t=W_{out} \cdot r_t$) and the actual control outputs $y_{\text {target}, t}$ recorded in the training data. By finding the optimal $W_{out}$ that minimizes the mean squared error between the predicted and true values, the reservoir network learns the complex nonlinear mapping from vehicle motion states (input) to precise control commands (output). The output weights $W_{out}$ are estimated using ridge regression (least squares with $L_2$ regularization), where $\beta$ is the regularization coefficient.

19 $$\begin{aligned} W_{out} = Y_{target} R^\top (R R^\top + \beta I)^{-1} \end{aligned}$$

This closed-form solution ensures a trade-off between effectively reducing training error and maintaining the model’s numerical stability. The regularization term $\beta$ effectively prevents overfitting and improves the model’s generalization ability. After training, the output weights $W_{out}$ and the final state $r_{N_{train}}$ are saved together for continuing the reservoir state updates during the subsequent validation phase.

Next, the trained RC network is validated on a target trajectory. The desired longitudinal acceleration, $a_{x,desire}$, and lateral acceleration, $a_{y,desire}$, are calculated from the desired trajectory and used as inputs in place of the future accelerations $a_x(t+dt)$ and $a_y(t+dt)$ from the training phase. By taking the first-order numerical derivative of the desired position coordinates $X(t), Y(t)$ with respect to time t, we obtain the desired velocity components in the inertial frame, $\dot{X}(t)$ and $\dot{Y}(t)$. The desired speed $v(t)$ is then calculated as $v(t) = \sqrt{\dot{X}(t)^2 + \dot{Y}(t)^2}$. To obtain the parameter describing the path’s curvature, the instantaneous curvature $K(t)$ of the trajectory is calculated:

20 $$\begin{aligned} K(t) = \frac{\dot{X}(t)\ddot{Y}(t) - \dot{Y}(t)\ddot{X}(t)}{(\dot{X}(t)^2 + \dot{Y}(t)^2)^{3/2}} \end{aligned}$$

**Fig. 4:**
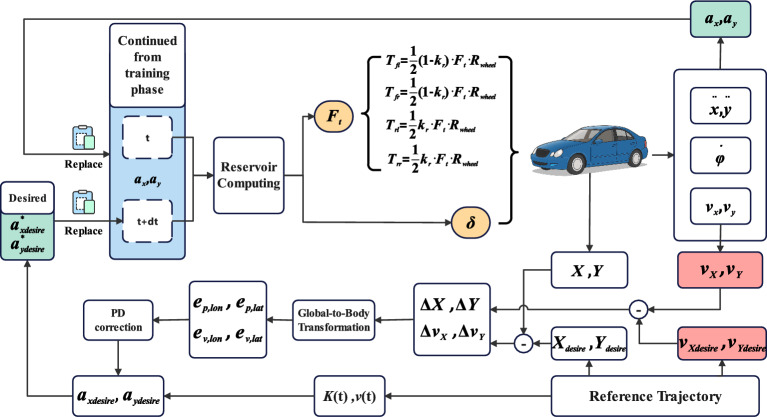
Validation phase.

The desired lateral acceleration is calculated based on the desired speed $v(t)$ and instantaneous curvature $K(t)$ as $a_{y,desire}(t) = v(t)^2 \cdot K(t)$, and the desired longitudinal acceleration is $a_{x,desire}(t) = \frac{dv(t)}{dt}$. Through these steps, the required desired longitudinal and lateral accelerations at each moment can be derived from a given time-varying desired trajectory^27^. To suppress trajectory drift caused by accumulated position errors, a closed-loop correction is applied to the desired accelerations, $a_{x,desire}$ and $a_{y,desire}$, at each control cycle $t_i$, as illustrated in Fig. 4. The correction process consists of three steps: First, the position tracking error $(\Delta X, \Delta Y)$ is computed in the global coordinate system. Second, the velocity error is calculated by transforming the vehicle’s body-frame velocity to the global frame and comparing it with the desired global velocity, yielding $(\Delta v_x, \Delta v_y)$. Third, these global-frame errors are projected onto the desired trajectory’s body frame using a rotation matrix based on the desired heading angle $\theta _{des}$, resulting in longitudinal errors $(e_{p,lon}, e_{v,lon})$ and lateral errors $(e_{p,lat}, e_{v,lat})$. A ’PD correction’ module, which is a PD controller, then calculates the longitudinal and lateral acceleration correction terms ($a_{x,corr}, a_{y,corr}$) based on these error components. The control law is as follows:

21 $$\begin{aligned} & a_{x,corr} = -K_{p,lon} \cdot e_{p,lon} - K_{d,lon} \cdot e_{v,lon} \end{aligned}$$

22 $$\begin{aligned} & a_{y,corr} = -K_{p,lat} \cdot e_{p,lat} - K_{d,lat} \cdot e_{v,lat} - K_{p,head} \cdot v_{des} \cdot e_{\psi } \end{aligned}$$

Here, $K_p$ and $K_d$ represent the proportional and derivative gains, respectively. The subscripts *lon* and *lat* denote the longitudinal and lateral directions, while *head* refers to the heading angle correction. The specific gains used in this study were set to $K_{p,lon} = K_{p,lat} = 4.0$, $K_{d,lon} = K_{d,lat} = 6.0$, and $K_{p,head} = 0.5$. $e_{\psi }$ is the heading angle error, and $v_{des}$ is the desired speed. By adding these correction terms to the original desired accelerations, we get the corrected values $a_{x,desire}^*$ and $a_{y,desire}^*$. As depicted in Fig. 4, the validation phase leverages the input vector structure established during training, which is composed of acceleration data at time $t$ and $t+dt$. For real-time control, the vehicle’s actual measured accelerations from the current moment, $a_x(t)$ and $a_y(t)$ (shown in green), replace the data in the $t$ slot of the training framework. Simultaneously, the corrected desired accelerations, $a_{x,desire}^*$ and $a_{y,desire}^*$, replace the data in the $t+dt$ slot. This substitution forms the final input vector $z_v$ for the validation phase:

23 $$\begin{aligned} z_v = [a_x(t), a_y(t), a_{x,desire}^*, a_{y,desire}^*]^\top \end{aligned}$$

As shown in Fig. 4, the validation process begins with the reservoir’s internal state continued from the final state of the training phase ($r_{N_{train}}$). This input vector $z_v$ is then fed into the RC network to update the state and compute the required control commands. The total longitudinal force $F_t$ output by the RC network is distributed to the front and rear axles following the dynamic allocation strategy detailed in the Dynamics modeling subsection, with each axle’s force then equally split between the left and right wheels. This completes the closed-loop control cycle.

### Trajectory tracking performance validation and comparison

The performance of the RC network is sensitive to its hyperparameters. We selected six core hyperparameters for optimization as they directly govern the physical characteristics of the reservoir dynamics: the spectral radius $\rho$ and leak rate $\alpha$ determine the fading memory timescale; the input weight magnitude $\gamma$ and bias $k_b$ control the nonlinearity regime; and the sparsity $k$ and regularization coefficient $\beta$ balance model complexity and generalization. Notably, the reservoir size was fixed at $n=200$ as a structural parameter, balancing computational efficiency with sufficient dimensionality for vehicle dynamics embedding.

We selected the double-lane-change tracking task at 60 km/h as the optimization scenario, as this task can thoroughly test the controller’s transient response and rapid steering capabilities at high speed. The optimization process employed MATLAB’s surrogateopt solver to jointly optimize these six core hyperparameters with the goal of minimizing trajectory tracking error. Let the hyperparameter vector be:

24 $$\begin{aligned} \theta = [\rho , \gamma , \alpha , \log _{10}\beta , k, k_b]^\top \end{aligned}$$

The search space was defined as: $0.1 \le \rho \le 1.2$, $0.1 \le \gamma \le 1.0$, $0 \le \alpha \le 0.99$, $-6 \le \log _{10}\beta \le 2$, $0.05 \le k \le 0.95$, $0 \le k_b \le 2$.

**Fig. 5:**
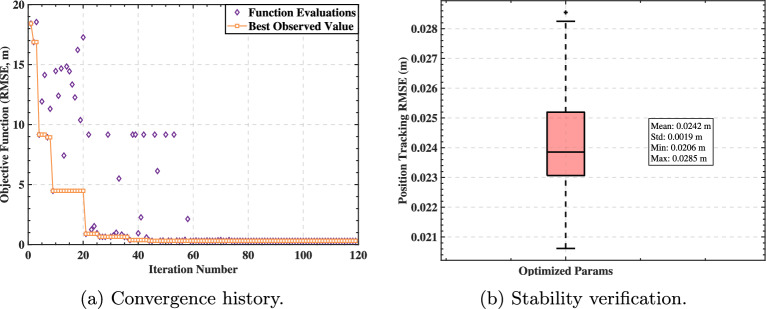
Optimization convergence and stability verification. (**a**) The objective function (RMSE) decreases rapidly and converges within 40 iterations. (**b**) The boxplot of tracking RMSE across 50 independent runs with optimized hyperparameters demonstrates low variance and high robustness.

The objective function was defined as the mean RMSE over multiple independent trials to explicitly account for the stochastic nature of RC initialization. For each candidate hyperparameter set sampled by the solver, we conducted 20 independent training-validation runs with different random seeds for network initialization. The objective value returned to the optimizer was the average of the best 6 runs, ensuring the optimization sought parameters with high potential performance rather than those dependent on lucky initialization. As shown in Fig. 5a, the best observed value converged rapidly within approximately 40 iterations out of a 120-iteration budget, while the optimizer continued to explore the parameter space to ensure global optimality. Furthermore, Fig. 5b illustrates the stability of the final optimized model: across 50 independent runs with random initializations, the tracking performance distribution is tightly clustered (Standard Deviation $\approx$ 1.9 mm), confirming that the proposed method is robust and reproducible.

The final optimal hyperparameter vector obtained was:

25 $$\begin{aligned} \theta ^* = [0.919, 0.116, 0.015, -1.96, 0.29, 1.99] \end{aligned}$$

After training the RC network with the optimal hyperparameters, a series of trajectory tracking experiments were conducted to compare the proposed RC+PD control strategy with the following two methods: (1) A pure RC controller (RC without PD): This serves as an ablation study to evaluate the role of the PD feedback component. (2) NMPC: This serves as a high-precision benchmark, implemented using the CasADi framework with the IPOPT solver, to gauge the performance of the RC+PD method in terms of tracking accuracy and computational efficiency.

**Fig. 6:**
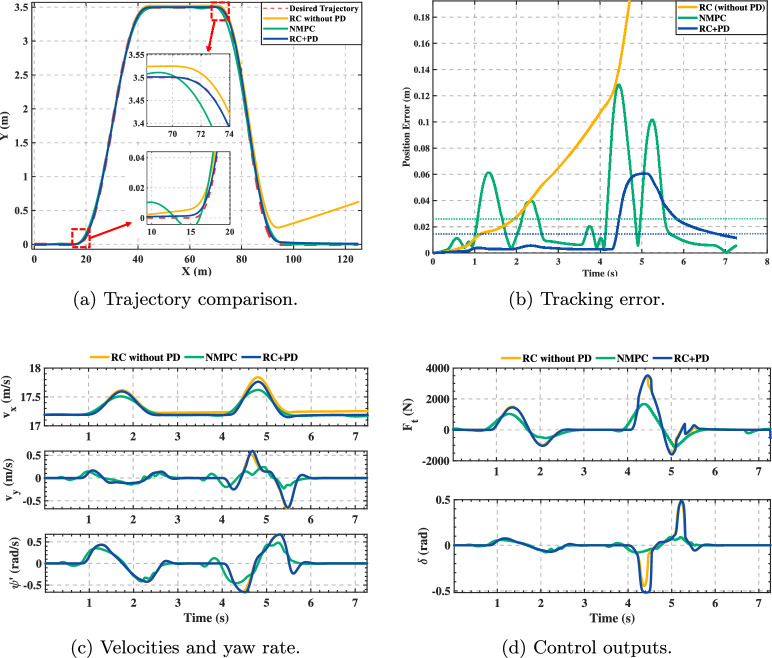
Double-lane-change trajectory tracking.

The tracking results for the double-lane-change trajectory are shown in Fig. 6. Subplot (a) demonstrates that the proposed RC+PD controller achieves superior path conformity compared to NMPC, while the pure RC controller exhibits significant deviation. The zoomed-in insets reveal critical transient behavior: during both lane changes (10–18 m and 68–74 m), RC+PD maintains tight tracking, NMPC shows lag and slight inner-cutting, and pure RC loses tracking capability. Subplot (b) quantifies this: RC+PD error remains low and bounded with rapid convergence after peaks (around 5 s), NMPC exhibits larger oscillations, while pure RC diverges after 4 s, confirming the necessity of PD feedback. Subplot (c) shows all methods maintain reasonable dynamic states, with RC+PD exhibiting the smoothest yaw rate response. Critically, subplot (d) reveals that RC+PD produces smooth, chatter-free control signals $F_t$ and $\delta$ throughout, whereas NMPC exhibits high-frequency oscillations detrimental to actuator longevity and ride comfort.

**Fig. 7:**
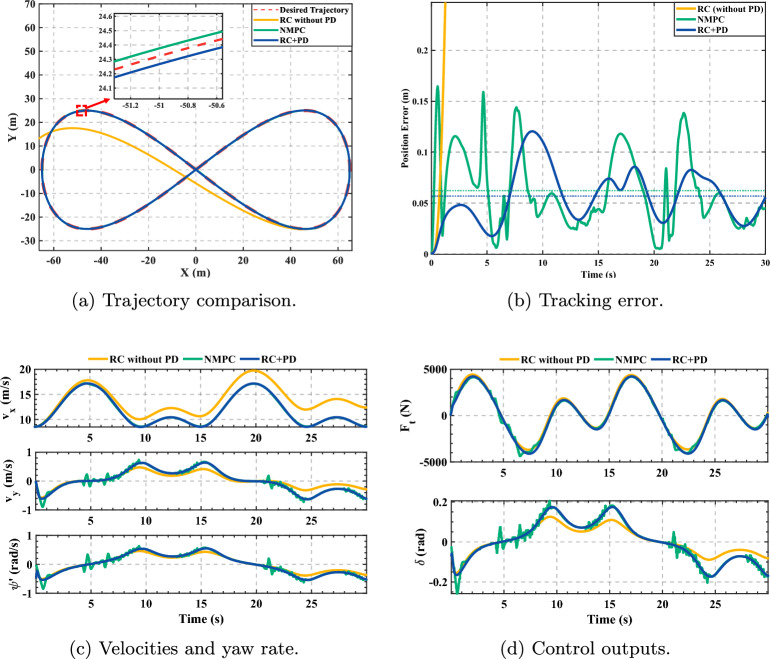
Figure-eight trajectory tracking.

To further evaluate generalization beyond the optimization scenario, the figure-eight trajectory was tested, which challenges long-term stability through sustained large-curvature turns. Figure 7 reveals a critical performance hierarchy. Subplot (a) shows that while RC+PD and NMPC successfully complete the closed loop, the pure RC controller fails catastrophically. The zoomed-in inset pinpoints failure initiation at the first sharp turn, where the trajectory deviates sharply. Subplot (b) quantifies this divergence, with error growing unboundedly shortly thereafter, confirming that PD feedback is essential for suppressing error accumulation in extended tasks.

Between the two successful controllers, NMPC achieves marginally lower steady-state error (smaller periodic peaks in subplot (b)), yet this comes at the cost of control quality. While subplot (c) shows comparable velocity and yaw rate dynamics, subplot (d) exposes persistent high-frequency chatter in NMPC’s steering command $\delta$, contrasting sharply with RC+PD’s smooth outputs across both $F_t$ and $\delta$.

**Fig. 8:**
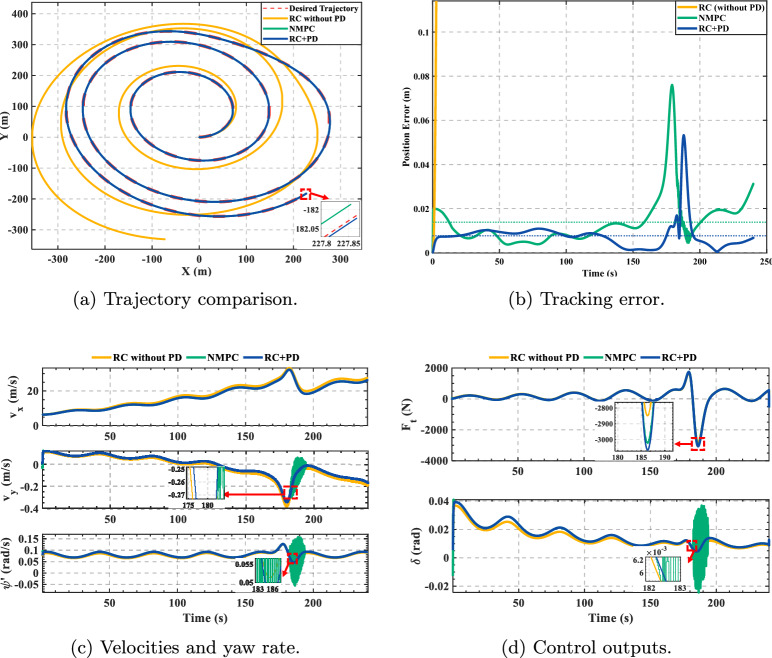
Rössler trajectory tracking.

Finally, a Rössler chaotic trajectory was introduced for tracking. This trajectory originates from a classic three-dimensional nonlinear dynamical model. Its core challenge lies in the path’s high degree of unpredictability and non-repetitive nature, making it an excellent benchmark for evaluating a controller’s generalization ability in a truly complex environment^28–31^. However, to ensure the test was physically feasible for a vehicle, we selected the “initial acceleration segment” of its two-dimensional projection. This segment retains the path’s nonlinear characteristics and sustained acceleration demands while avoiding the impractical dynamics of the full chaotic attractor. The trajectory coordinates were also scaled up to a 300 m x 300 m area. The test results are shown in Fig. 8.

Focusing on Fig. 8, the key observations are as follows. Overall, subplot (a) shows that both RC+PD and NMPC complete the tracking task, whereas the pure RC controller diverges at the outset. The RC+PD trajectory almost coincides with the reference curve, indicating high global fidelity. In the red boxed high-curvature segment of (a), RC+PD exhibits neither noticeable inner-cutting nor lag, while NMPC shows slight lag and inward deviation. This aligns with subplot (b): within the highlighted window (approximately $t\!\in \![175,190]\,$s), the peak error of RC+PD is distinctly lower and converges more rapidly back to a low steady level.

Cross-examining the vehicle states in subplot (c) clarifies the mechanism: in the same window, RC+PD maintains smooth variations in lateral velocity $v_y$ and yaw rate $\dot{\psi }$ with small ripples, whereas NMPC presents higher-frequency oscillations and transient amplification, suggesting a stronger sensitivity to the lateral–longitudinal coupling. The control signals in subplot (d) further corroborate this: NMPC’s steering command $\delta$ displays pronounced high-frequency chatter in the red box, accompanied by larger fluctuations in the traction/braking force $F_t$. In contrast, RC+PD generates smooth and bounded commands for both $F_t$ and $\delta$, which is consistent with its lower instantaneous error and faster recovery. Collectively, these results indicate that, under the most demanding segments of the Rössler trajectory, RC+PD suppresses state oscillations through realizable, smooth actuation, thereby achieving smaller peak errors and quicker convergence; the pure RC controller, lacking feedback, becomes unstable early on.

The quantitative performance comparison across all three trajectories is summarized in Table 1. The results demonstrate that the proposed RC+PD method achieves superior tracking accuracy while maintaining computational efficiency advantages. Specifically, on the double-lane-change trajectory, RC+PD outperforms NMPC in both accuracy (RMSE reduced by 41.8%) and efficiency (2.20$\times$ speedup). On the figure-eight trajectory, RC+PD achieves 12.7% lower RMSE than NMPC with a 1.90$\times$ speedup, while also producing smoother control signals. For the highly challenging Rössler chaotic trajectory, RC+PD attains remarkable centimeter-level precision (RMSE of 10.2 mm) compared to NMPC’s 17.6 mm, completing the task 1.71$\times$ faster. Notably, the pure RC controller exhibits catastrophic failure on the figure-eight and Rössler trajectories with RMSE exceeding tens of meters, confirming the indispensable role of PD feedback in ensuring closed-loop stability. Overall, the RC+PD framework consistently delivers superior accuracy and computational efficiency across diverse trajectory types, making it particularly suitable for practical autonomous driving applications.

**Table 1:** Quantitative performance comparison of three control methods across different trajectories.

Trajectory	Method	RMSE (m)	Time (s)	Speedup
Double-lane-change	RC+PD	0.0233	5.03	2.20$\times$
	NMPC	0.0400	11.07	–
	Pure RC	0.2905	6.03	–
Figure-eight	RC+PD	0.0627	14.74	1.90$\times$
	NMPC	0.0718	27.98	–
	Pure RC	Failed	15.04	–
Rössler	RC+PD	0.0102	105.59	1.71$\times$
	NMPC	0.0176	181.04	–
	Pure RC	Failed	105.77	–

## Robustness analysis

This section evaluates the controller’s stability under external disturbances and sensor uncertainties through robustness testing. To comprehensively assess the robustness characteristics, we conducted systematic tests on both the figure-eight and Rössler trajectories, which represent different dynamic challenges. The figure-eight trajectory provides periodic and repeatable conditions ideal for evaluating steady-state robustness, while the Rössler chaotic trajectory offers non-repetitive, unpredictable disturbances that test the controller’s generalization ability under highly complex conditions.

The disturbance was applied to the controller’s raw output commands $u(t)$:

26 $$\begin{aligned} u_{disturbed}(t) = u(t) + \lambda _d \cdot \sigma _u \cdot \mathcal {N}(0,1) \end{aligned}$$

Noise was applied to the raw acceleration signals $a(t)$, which serve as the controller’s input:

27 $$\begin{aligned} a_{noisy}(t) = a(t) + \lambda _n \cdot \sigma _a \cdot \mathcal {N}(0,1) \end{aligned}$$

**Fig. 9:**
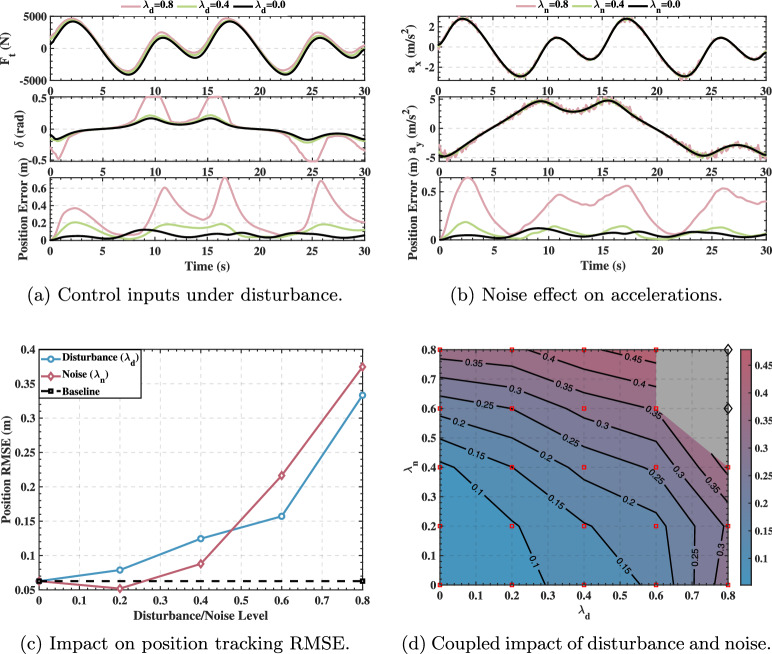
Comprehensive robustness analysis on the figure-eight trajectory under control disturbance and sensor noise.

In the models above, $u(t)$ and $a(t)$ are the original signals, respectively; $\lambda _d$ and $\lambda _n$ are adjustable coefficients for the disturbance and noise levels; $\sigma _u$ and $\sigma _a$ are the standard deviations of the corresponding historical signals; and $\mathcal {N}(0,1)$ represents a standard normal distribution. By independently adjusting the disturbance coefficient $\lambda _d$ and the noise coefficient $\lambda _n$, we can systematically evaluate the controller’s performance degradation under various levels of interference. The comprehensive impacts of these two types of interference on the controller’s tracking performance for the figure-eight trajectory, both individually and jointly, are shown in Fig. 9.

Figure 9 presents a comprehensive robustness analysis on the figure-eight trajectory. Subfigure (a) shows the time series of control inputs ($F_t$, $\delta$) and instantaneous position error under three representative control disturbance levels: $\lambda _d=0.0$ (no disturbance, black), $\lambda _d=0.4$ (moderate disturbance, green), and $\lambda _d=0.8$ (high disturbance, pink). As disturbance intensity increases, the control commands exhibit progressively larger random fluctuations, and the instantaneous tracking error escalates correspondingly—from approximately 0.05 m at baseline to around 0.2 m at moderate disturbance, and further to 0.6–0.7 m under high disturbance. Subfigure (b) displays the measured accelerations ($a_x$, $a_y$) and instantaneous position error under three sensor noise levels: $\lambda _n=0.0$, $\lambda _n=0.4$, and $\lambda _n=0.8$. The acceleration signals demonstrate increasing noise contamination with higher $\lambda _n$, accompanied by deteriorating position tracking accuracy—from baseline performance to approximately 0.2 m error at $\lambda _n=0.4$, and up to 0.65 m at $\lambda _n=0.8$.

Subfigure (c) quantitatively compares the impact of disturbance and noise on position tracking RMSE across the full test range (0–80%). The baseline RMSE under ideal conditions (black dashed line) is approximately 0.062 m. For control command disturbance (blue curve with circles), the RMSE exhibits a nearly monotonic increase from about 0.065 m at zero disturbance to approximately 0.33 m at 80% disturbance, indicating that control disturbances consistently degrade tracking performance. In contrast, sensor measurement noise (red curve with diamonds) reveals a noteworthy non-monotonic phenomenon: the RMSE initially decreases from the baseline value to a minimum of approximately 0.05 m at around 20% noise level, then progressively increases to about 0.37 m at 80% noise. This demonstrates that moderate sensor noise can paradoxically enhance tracking accuracy, exhibiting a noise-assisted performance enhancement effect.

Subfigure (d) presents a systematic evaluation of the coupled impact through a two-dimensional parameter sweep spanning $\lambda _d \in [0, 0.8]$ and $\lambda _n \in [0, 0.8]$. The color gradient (from blue indicating low RMSE to red/gray indicating high RMSE) and contour lines quantitatively map the tracking error across the disturbance-noise space. Red square markers denote successful tracking trials (RMSE < 0.5 m), while black diamond markers indicate tracking failures (RMSE > 0.5 m), with the gray shaded region representing the failure zone. This analysis reveals that the controller maintains robust performance across a broad operational envelope, successfully tracking under combined disturbances up to approximately $\lambda _d=0.8$ with $\lambda _n \le 0.4$, or $\lambda _n=0.6$ with $\lambda _d \le 0.6$. However, tracking fails when both interference sources reach high levels simultaneously ($\lambda _d \ge 0.8$ and $\lambda _n \ge 0.6$), demonstrating a synergistic amplification effect where concurrent high-intensity disturbances breach the system’s robustness boundary. This nonlinear interaction indicates that the combined impact of dual interference sources exceeds simple linear superposition.

**Fig. 10:**
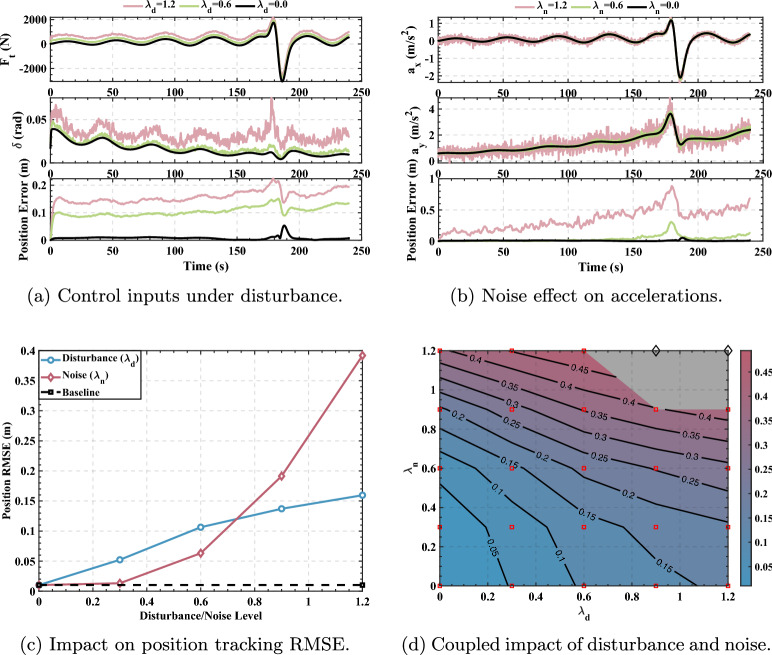
Comprehensive robustness analysis on the Rössler chaotic trajectory under control disturbance and sensor noise.

To further validate the robustness findings under more challenging conditions, similar comprehensive tests were conducted on the Rössler chaotic trajectory. The controller’s baseline performance on this trajectory under ideal conditions resulted in an RMSE of 0.0102 m, which serves as the benchmark for the subsequent robustness evaluations. The comprehensive robustness analysis results for the Rössler trajectory are presented in Fig. 10.

Figure 10 presents the robustness analysis on the Rössler chaotic trajectory using the same analytical framework. Subfigures (a) and (b) demonstrate that under representative disturbance levels ($\lambda _d \in \{0.0, 0.6, 1.2\}$ and $\lambda _n \in \{0.0, 0.6, 1.2\}$), the instantaneous tracking error increases from baseline (approximately 0.01 m) to approximately 0.1–0.15 m at moderate levels and 0.15–0.6 m at high levels, confirming progressive performance degradation with increasing interference intensity.

Subfigure (c) reveals critical differences compared to the figure-eight trajectory. The baseline RMSE is 0.0102 m. Control disturbances (blue curve) exhibit monotonic degradation from 0.01 m to 0.16 m at 120% disturbance. Notably, sensor noise (red curve) demonstrates a subtle non-monotonic trend: RMSE marginally increases to approximately 0.015 m at 30% noise before progressively rising to 0.39 m at 120% noise. This indicates that the noise-assisted enhancement effect, while present, is significantly weaker on the chaotic trajectory compared to the periodic figure-eight path.

Subfigure (d) maps the failure boundaries across $\lambda _d, \lambda _n \in [0, 1.2]$. The controller maintains robust tracking (red squares) across a broader operational envelope than the figure-eight case, successfully handling combined disturbances up to $\lambda _d=1.2$ with $\lambda _n \le 0.9$, or $\lambda _n=1.2$ with $\lambda _d \le 0.6$. Tracking failures (black diamonds, gray region) occur when $\lambda _d \ge 1.2$ and $\lambda _n \ge 1.2$, or $\lambda _n \ge 1.2$ with $\lambda _d \ge 0.9$. This extended robustness envelope, despite the trajectory’s chaotic nature, demonstrates the controller’s superior generalization capability across fundamentally different dynamic conditions.

## Discussion

### Comprehensive evaluation of control performance

To address the trajectory tracking challenges caused by the strong nonlinearity and coupling of vehicle lateral and longitudinal dynamics under high-dynamic and complex conditions, this paper proposed and validated a control framework combining RC-based inverse dynamics feedforward with PD feedback compensation. Across a series of tracking tasks ranging from conventional to extreme, the proposed RC+PD controller consistently achieved centimeter-level tracking accuracy. In a direct comparison with a fully optimized NMPC benchmark (Table 1), our method demonstrated significant advantages across all tested trajectories. On the double-lane-change task, RC+PD achieved 41.8% lower tracking error than NMPC with 2.20$\times$ speedup. On the figure-eight trajectory, RC+PD outperformed NMPC by 12.7% in accuracy while being 1.90$\times$ faster and producing smoother control signals. Most remarkably, on the challenging Rössler chaotic trajectory, RC+PD achieved 10.2 mm RMSE (42.0% better than NMPC) with 1.71$\times$ computational efficiency gain. Overall, the RC+PD framework consistently delivers superior accuracy and efficiency across diverse conditions. Furthermore, the ablation study comparison with the pure RC controller clearly showed that the absence of PD feedback leads to catastrophic divergence in long-duration or high-curvature tasks, highlighting the critical role of PD closed-loop correction in guaranteeing system stability and final accuracy.

### Generalization capability and trajectory-dependent noise response

The systematic validation across three distinct trajectories demonstrates robust generalization beyond the training distribution. The controller, optimized exclusively on the double-lane-change scenario, successfully tracks both the periodic figure-eight and the aperiodic Rössler chaotic trajectory without retraining, achieving centimeter-level precision across all conditions. This generalization capability stems from the RC network’s ability to learn high-dimensional nonlinear embeddings of the vehicle’s coupled dynamics during offline training on diverse excitation data, enabling accurate inverse mapping across previously unseen operating regimes.

The robustness analysis reveals a counterintuitive phenomenon that challenges conventional control wisdom. Moderate sensor measurement noise exhibits a trajectory-dependent performance enhancement effect: on the figure-eight trajectory, RMSE decreases from 0.062 m to a minimum of 0.05 m at $\lambda _n \approx 0.2$, representing a 19% accuracy improvement. However, this effect significantly weakens on the Rössler chaotic trajectory, where RMSE shows only marginal variation (from 0.0102 m to approximately 0.015 m) before monotonically increasing. Critically, control command disturbances exhibit no beneficial range on either trajectory, consistently degrading performance in a near-monotonic fashion.

This trajectory-dependent noise response may arise from the interplay between reservoir dynamics and trajectory predictability. For periodic trajectories, moderate noise may induce stochastic resonance within the reservoir’s high-dimensional state space^32,33^, where random perturbations enhance the system’s sensitivity to underlying periodic patterns by preventing premature convergence to local attractors. In contrast, the Rössler trajectory’s inherent aperiodicity and high complexity diminish this resonance effect, as the absence of stable periodic structures reduces opportunities for noise-enhanced pattern detection. This contrasts sharply with model-based controllers like NMPC, which operate within precisely defined solution spaces where noise universally degrades performance^34^. The observed phenomenon suggests potential for active noise injection strategies to systematically exploit this effect in future designs, though practical implementation requires careful calibration to trajectory characteristics.

### Critical role of PD feedback and failure boundary characterization

The ablation study with pure RC (without PD feedback) provides unequivocal evidence of PD’s indispensability. While pure RC successfully tracks the short-duration double-lane-change, it fails catastrophically on extended trajectories—diverging completely on both the figure-eight and Rössler paths. This stark contrast reveals that feedforward inverse dynamics alone cannot compensate for cumulative errors or unmodeled disturbances inherent in real-world operation. The PD component fulfills three critical functions: (1) suppressing error accumulation through proportional correction of position deviations, (2) damping transient oscillations via derivative action on velocity errors, and (3) providing robustness against model uncertainties and external disturbances.

The synergy between RC feedforward and PD feedback represents a paradigm shift in data-driven control architecture. The RC network encodes complex lateral-longitudinal coupling dynamics offline through high-dimensional nonlinear mapping, enabling real-time inverse computation via a single matrix-vector multiplication. This feedforward path proactively generates control commands aligned with desired dynamics. Simultaneously, the PD feedback loop closes the system online, stabilizing against deviations and ensuring bounded tracking error. This hybrid design achieves what neither component accomplishes independently—matching or exceeding NMPC’s tracking precision while delivering computational efficiency orders of magnitude higher.

The comprehensive robustness characterization through two-dimensional parameter sweeps clearly delineates operational boundaries. On the figure-eight trajectory, stable tracking persists up to $\lambda _d=0.8, \lambda _n=0.4$, with failure occurring when both exceed $\lambda _d \ge 0.8, \lambda _n \ge 0.6$. The Rössler trajectory exhibits a surprisingly broader envelope ($\lambda _d=1.2, \lambda _n=0.9$ or $\lambda _n=1.2, \lambda _d=0.6$), despite its chaotic nature. This counter-intuitive finding suggests that the learned inverse dynamics exhibit greater robustness to high-frequency disturbances than to long-term drift in repetitive patterns. The identified synergistic amplification effect (where combined disturbances breach robustness boundaries earlier than single-factor extrapolation would predict) provides crucial design guidance for safety-critical deployment.

### Comparative analysis with deep recurrent neural networks

While deep recurrent neural networks such as LSTM have achieved success in sequence modeling, the choice of Reservoir Computing for vehicle inverse dynamics in this study is motivated by fundamental differences in training paradigms and generalization characteristics.

LSTM networks, trained via iterative gradient descent (BPTT), typically function as *interpolators* that learn mappings predominantly within the convex hull of training data^35^. When deployed on aggressive maneuvers requiring control magnitudes beyond the training distribution, LSTMs may exhibit *distribution lock-in*—producing conservative outputs bounded by the observed range. In contrast, RC employs a closed-form ridge regression solution for its linear readout layer, which inherently possesses *extrapolation capability*, allowing the linear mapping to extend naturally beyond training boundaries.

Beyond generalization, the computational disparity is substantial. LSTM training relies on iterative backpropagation through time, which is computationally expensive. Conversely, RC’s training is reduced to a linear least-squares problem, enabling extremely rapid, non-iterative learning. This efficiency is not merely a convenience but a prerequisite for potential online learning and rapid adaptation in changing vehicle environments.

Consequently, RC offers a dual advantage for trajectory tracking control: its linear extrapolation capability handles out-of-distribution dynamics inherent in limit handling, while its computational efficiency supports real-time deployment and future online adaptation. Appendix C provides empirical validation of these architectural benefits.

## Conclusion

This paper proposes an RC-based inverse dynamics control framework combined with PD feedback for autonomous vehicle trajectory tracking. The RC network implicitly decouples lateral-longitudinal dynamics through offline learning, while PD feedback ensures closed-loop stability. Validation on double-lane-change, figure-eight, and Rössler chaotic trajectories demonstrates 12.7%–42.0% accuracy improvement and 1.71$\times$–2.20$\times$ speedup over NMPC, with centimeter-level precision (10.2 mm RMSE) maintained on unseen chaotic paths. Robustness analysis characterizes failure boundaries under combined disturbances and reveals a trajectory-dependent noise-enhancement phenomenon.

Current limitations include simulation-only validation, stationary vehicle parameters, and the focus on mid-to-high speed dynamics (40–70 km/h). Future work will address real-vehicle implementation and online incremental learning for parameter adaptation, leveraging RC’s efficient closed-form retraining.

Beyond vehicle control, the proposed paradigm may extend to robotic arm motion^36^, where RC-based inverse dynamics could elevate control from kinematic velocity planning to dynamic torque generation with *O*(1) inference complexity, and to large-scale cluster systems^37^, where RC’s fading memory enables predictive state estimation during communication disruptions caused by cyber-attacks.

## Figures and Tables

**Fig. 11:**
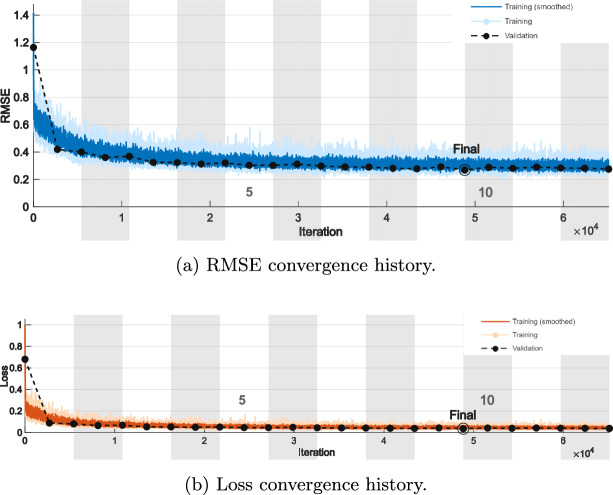
Training convergence history of the LSTM network.

**Fig. 12:**
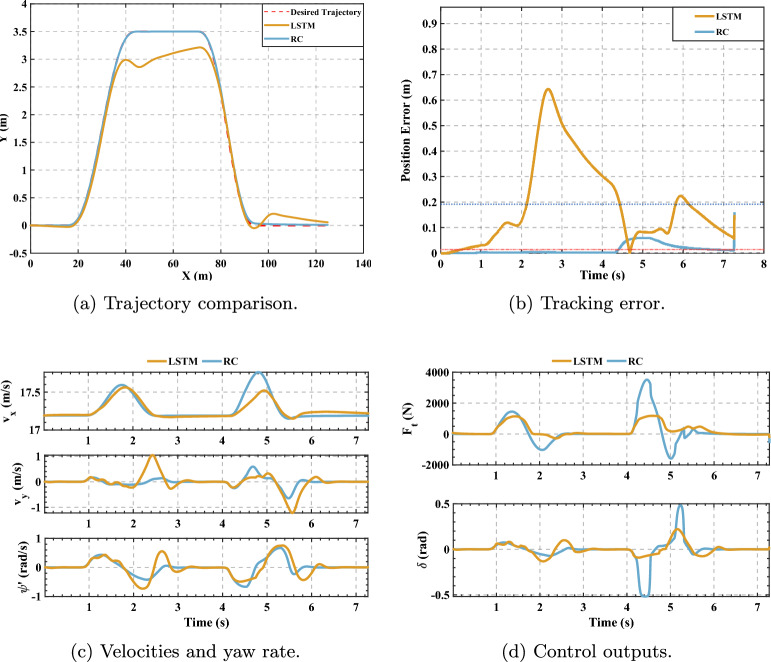
Performance comparison between RC and LSTM controllers on the double-lane-change trajectory.

**Fig. 13:**
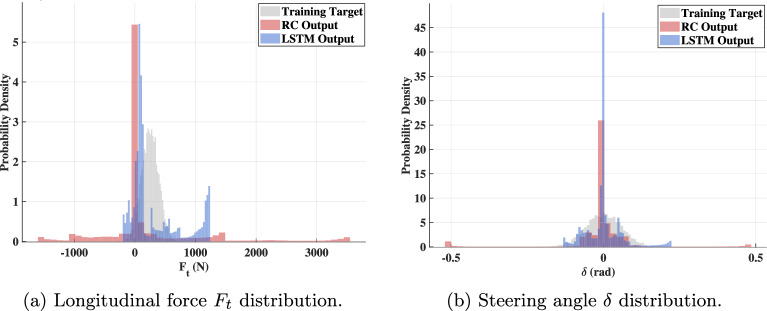
Output distribution comparison among training targets, RC, and LSTM. The grey region indicates training data distribution; red and blue histograms show RC and LSTM outputs during validation, respectively.

## Data Availability

The datasets generated during the current study are available from the corresponding author on reasonable request.

## Appendix A: Simulation parameters

The simulation parameters used in this study are listed in Table 2.

**Table 2:** Simulation parameters used in the vehicle dynamics model and controller validation.

Parameter	Symbol	Value	Unit
Vehicle mass	*m*	1460	kg
Distance from CG to front axle	$l_f$	1.23	m
Distance from CG to rear axle	$l_r$	1.51	m
Moment of inertia	$I_z$	2269	$\hbox {kg}\cdot \hbox {m}^{2}$
Height of center of gravity (CG)	$h_{cg}$	0.5	m
Road friction coefficient	$\mu$	0.7–1.0	-
Air drag coefficient	$k_h$	0.35	kg/m
Tire stiffness factor (front/rear)	$B_f, B_r$	12	-
Tire shape factor (front/rear)	$C_f, C_r$	1.5	-
Front wheel tire peak factor	$D_{0f}$	4000	N
Rear wheel tire peak factor	$D_{0r}$	3500	N
Tire curvature factor (front/rear)	$E_f, E_r$	0.9	-

## Appendix B: Derivation of coupling effect sensitivity equations

This appendix details the derivation of the sensitivity equations describing the coupling effects between the vehicle’s lateral and longitudinal dynamics.

### B.1 Sensitivity of longitudinal acceleration to steering angle

Starting from the longitudinal dynamics equation (Equation (1)), with $a_x = \ddot{x} - \dot{y}\dot{\phi }$:

B1 $$\begin{aligned} m a_x = F_{xf}\cos \delta - F_{yf}\sin \delta + F_{xr} - k_h\dot{x}^2 \end{aligned}$$

To find the sensitivity of $a_x$ to $\delta$, we first differentiate the complete equation with respect to $\delta$, then apply small-angle approximations. The longitudinal forces $F_{xf}$ and $F_{xr}$ are primarily determined by the powertrain/brake commands and are treated as independent of $\delta$. However, the lateral force $F_{yf}$ depends on the tire slip angle $\alpha _f$, which is itself a function of $\delta$.

The front tire slip angle is defined as:

B2 $$\begin{aligned} \alpha _f = \delta - \arctan \left( \frac{\dot{y} + l_f \dot{\phi }}{v_x}\right) \end{aligned}$$

For small angles, this simplifies to $\alpha _f \approx \delta - (\dot{y} + l_f \dot{\phi })/v_x$. Since the vehicle’s kinematic states ($\dot{y}$, $\dot{\phi }$, $v_x$) are independent of the instantaneous steering input $\delta$, we have $\frac{\partial \alpha _f}{\partial \delta } = 1$.

Under the linearized tire model near $\alpha = 0$, the lateral force is proportional to the slip angle: $F_{yf} \approx C_{f,eff} \alpha _f$, where $C_{f,eff} = B_f C_f D_f$ is the effective cornering stiffness. Applying the chain rule:

B3 $$\begin{aligned} \frac{\partial F_{yf}}{\partial \delta } = \frac{\partial F_{yf}}{\partial \alpha _f} \cdot \frac{\partial \alpha _f}{\partial \delta } = C_{f,eff} \cdot 1 = C_{f,eff} \end{aligned}$$

With this result established, we now differentiate the original equation term by term:

B4 $$\begin{aligned} m \frac{\partial a_x}{\partial \delta } = \frac{\partial }{\partial \delta }(F_{xf}\cos \delta ) - \frac{\partial }{\partial \delta }(F_{yf}\sin \delta ) \end{aligned}$$

Applying the product rule to each term:

B5 $$\begin{aligned} m \frac{\partial a_x}{\partial \delta } = -F_{xf}\sin \delta - \left( \frac{\partial F_{yf}}{\partial \delta }\sin \delta + F_{yf}\cos \delta \right) \end{aligned}$$

Substituting $$\frac{\partial F_{yf}}{\partial \delta } \approx C_{f,eff}$$:

B6 $$\begin{aligned} m \frac{\partial a_x}{\partial \delta } = -F_{xf}\sin \delta - C_{f,eff}\sin \delta - F_{yf}\cos \delta \end{aligned}$$

Now applying small-angle approximations ($\sin \delta \approx \delta$, $\cos \delta \approx 1$):

B7 $$\begin{aligned} m \frac{\partial a_x}{\partial \delta } \approx -F_{xf}\delta - C_{f,eff}\delta - F_{yf} \end{aligned}$$

Rearranging gives Equation (13):

B8 $$\begin{aligned} \frac{\partial a_x}{\partial \delta } = \frac{-(F_{xf}\delta + F_{yf} + C_{f,eff}\delta )}{m} \end{aligned}$$

### B.2 Sensitivity of lateral acceleration to longitudinal force

We analyze the steady-state turning condition. Starting from the lateral dynamics equation (Equation (2)) and the yaw dynamics equation (Equation (3)), we apply small-angle approximations ($\sin \delta \approx \delta$, $\cos \delta \approx 1$):

B9 $$\begin{aligned} m(\ddot{y} + \dot{x}\dot{\phi })&= F_{xf}\delta + F_{yf} + F_{yr} \end{aligned}$$

B10 $$\begin{aligned} I_z\ddot{\phi }&= l_f(F_{xf}\delta + F_{yf}) - l_r F_{yr} \end{aligned}$$

In steady-state turning, the yaw acceleration vanishes ($\ddot{\phi } = 0$) and the lateral acceleration is $a_y = \ddot{y} + \dot{x}\dot{\phi }$. This yields the force and moment balance equations:

B11 $$\begin{aligned} m a_y&= F_{yf} + F_{yr} + F_{xf}\delta \end{aligned}$$

B12 $$\begin{aligned} 0&= l_f(F_{yf} + F_{xf}\delta ) - l_r F_{yr} \end{aligned}$$

Solving the moment equation for $F_{yr}$ yields $F_{yr} = \frac{l_f}{l_r}(F_{yf} + F_{xf}\delta )$. Substituting this into the force equation:

B13 $$\begin{aligned} m a_y = F_{yf} + \frac{l_f}{l_r}(F_{yf} + F_{xf}\delta ) + F_{xf}\delta = \frac{L}{l_r}(F_{yf} + F_{xf}\delta ) \end{aligned}$$

Rearranging for $$F_{yf}$$:

B14 $$\begin{aligned} F_{yf} = \frac{l_r}{L} m a_y - F_{xf}\delta \end{aligned}$$

We now derive an alternative expression for $F_{yf}$ using the linearized tire model. From the slip angle definition established earlier, the front tire slip angle is:

B15 $$\begin{aligned} \alpha _f = \delta - \frac{\dot{y} + l_f \dot{\phi }}{v_x} \end{aligned}$$

Assuming the vehicle side-slip angle $\beta = \dot{y}/v_x$ is negligible for this sensitivity analysis, this simplifies to $\alpha _f \approx \delta - l_f \dot{\phi }/v_x$. Under the linearized tire model, the front lateral force becomes:

B16 $$\begin{aligned} F_{yf} \approx C_{f,eff} \alpha _f = C_{f,eff}\left( \delta - \frac{l_f \dot{\phi }}{v_x}\right) \end{aligned}$$

In steady-state turning, the vehicle follows a circular path with constant yaw rate $\dot{\phi }$. The centripetal acceleration required for circular motion is $a_y = v_x \dot{\phi }$, which gives $\dot{\phi } = a_y / v_x$. Substituting this relation:

B17 $$\begin{aligned} F_{yf} \approx C_{f,eff}\delta - \frac{C_{f,eff} l_f a_y}{v_x^2} \end{aligned}$$

Equating the two expressions for $F_{yf}$:

B18 $$\begin{aligned} \frac{l_r}{L} m a_y - F_{xf}\delta = C_{f,eff}\delta - \frac{C_{f,eff} l_f a_y}{v_x^2} \end{aligned}$$

Grouping terms with $a_y$:

B19 $$\begin{aligned} a_y \left( \frac{m l_r}{L} + \frac{C_{f,eff} l_f}{v_x^2} \right) = (C_{f,eff} + F_{xf})\delta \end{aligned}$$

Differentiating with respect to the total longitudinal force $F_t$, and noting that $F_{xf} = (1-k_r)F_t$ (so $\frac{\partial F_{xf}}{\partial F_t} = 1-k_r$), while treating $C_{f,eff}$ and $\delta$ as constants for this partial derivative:

B20 $$\begin{aligned} \frac{\partial a_y}{\partial F_t} \left( \frac{m l_r}{L} + \frac{C_{f,eff} l_f}{v_x^2} \right) = \delta (1-k_r) \end{aligned}$$

This yields Equation (14):

B21 $$\begin{aligned} \frac{\partial a_y}{\partial F_t} = \frac{\delta (1 - k_r)}{\frac{ml_r}{L} + \frac{C_{f,eff}l_f}{v_x^2}} \end{aligned}$$

## Appendix C: Comparative analysis with LSTM

This appendix presents a comparative evaluation between the proposed RC-based inverse dynamics model and a standard LSTM network, analyzing their performance characteristics and generalization capabilities.

### C.1 Experimental setup

To ensure a rigorous comparison, the LSTM model was trained and validated under conditions identical to the RC framework:

- **Dataset:** Both models utilized the same training dataset generated from the 3-DOF vehicle model with identical input features ($[a_x(t), a_y(t), a_x(t+dt), a_y(t+dt)]$) and target outputs ($[F_t, \delta ]$). While the raw control inputs were sampled from $F_t \sim U(-3000, 3000) + 250$ N as described in the Inverse dynamics model construction and control strategy subsection, the moving average smoothing significantly compresses the effective distribution, yielding training targets concentrated within $F_t \in [-265, 722]$ N and $\delta \in [-0.21, 0.20]$ rad.
- **Control Architecture:** The LSTM replaced the RC network within the exact same closed-loop control structure, including the same PD feedback gains.
- **LSTM Configuration:** The LSTM network consisted of a hidden layer with 64 units, followed by a fully connected layer. The sequence length was set to 100 time steps (100 ms) to capture sufficient dynamic history. The model was implemented using the MATLAB Deep Learning Toolbox and trained using the Adam optimizer with a learning rate of 0.001. To prevent overfitting, a dropout rate of 0.2 was applied, and early stopping was employed with a patience of 6 validation checks. The maximum number of epochs was set to 20.
- **Implementation Details:** Both input and target data were standardized (zero mean, unit variance). During validation, the LSTM operated in stateful mode—performing single-step inference while preserving hidden states—to replicate real-time control constraints and minimize latency.

### C.2 Results and analysis

The training convergence history of the LSTM network is illustrated in Figure 11. The alternating grey and white vertical bands indicate successive epochs, with the labels “5” and “10” marking the corresponding epoch indices. The solid black markers trace the validation metric, and the circled point annotated as “Final” denotes the epoch at which the minimum validation RMSE is achieved; the network parameters at this point are used for all subsequent evaluations. The curves exhibit a rapid initial decrease in prediction error and loss, followed by a slow approach to a plateau, indicating stable convergence.

The comparative results on the double-lane-change trajectory are presented in Figure 12.

**Key Observations:**

1. **Dynamic Responsiveness:** As shown in Figure 12d, the RC controller exhibits aggressive control actions with $F_t$ reaching $+3500$ N and $-2000$ N, while the LSTM controller produces conservative outputs confined to $\approx \pm 1500$ N.
2. **Tracking Accuracy:** The LSTM suffers from significant tracking lag (Figure 12c), yielding RMSE of 0.257 m—an order of magnitude higher than RC’s 0.023 m.
3. **Computational Efficiency:** Table 3 highlights the efficiency gap: RC’s closed-form training completes in 0.20 s versus LSTM’s 1047 s.

### C.3 Distribution analysis

To investigate the underlying causes of the observed performance disparity, we analyze the output control distributions of both controllers relative to the training targets (Figure 13).

The distributions reveal distinct behaviors:

- **LSTM (blue):** The output distribution closely aligns with the training targets (grey), with standard deviations of 426 N for $F_t$ and 0.059 rad for $\delta$ (ratios of 3.0$\times$ and 1.0$\times$ relative to training data, respectively). The control outputs remain largely confined within the training distribution envelope.
- **RC (red):** The output distribution extends significantly beyond training boundaries, with $F_t$ ranging from $-1590$ to $+3515$ N (standard deviation 839 N, 5.9$\times$ training) and $\delta$ spanning $\pm 0.52$ rad (standard deviation 0.12 rad, 2.0$\times$ training).

These empirical results align with the theoretical analysis of generalization characteristics. The LSTM, operating as a nonlinear interpolator, exhibits limited capacity to generate control magnitudes beyond its training experience. Conversely, the RC’s linear readout layer demonstrates the capability to extrapolate the learned inverse dynamics relationship, generating scaled control signals necessary for the out-of-distribution demands of the double-lane-change maneuver.

**Table 3:** Computational efficiency and performance comparison. All computations were executed on an Intel Core i9-14900K processor.

Method	Training method	Training time (s)	RMSE (m)
**RC (Ours)**	Ridge Regression	**0.20**^*^	**0.023**
LSTM	BPTT (20 epochs)	1047^**^	0.257

^*^Refers to the readout weight calculation step. State harvesting takes an additional 7.32 s.

^**^Actual training time for 12 epochs (early stopping triggered).

In conclusion, the comparative analysis highlights two key advantages of the RC architecture for this application: (1) the linear readout mechanism enables effective extrapolation for high-dynamic control outside the training distribution, and (2) the closed-form solution offers orders-of-magnitude faster training.
